# Design and Refractive Index Sensing Investigation of a Multi-Mode Switchable VO_2_-Graphene Terahertz Absorption Device

**DOI:** 10.3390/mi17101132

**Published:** 2026-09-29

**Authors:** Siwen Zhang, Jun Zhu, Zao Yi, Sohail Ahmad, Shicai Xu, Aiqing Li, Hailiang Li

**Affiliations:** 1 Joint Laboratory for Extreme Conditions Matter Properties, School of Mathematics and Science, Southwest University of Science and Technology, Mianyang 621010, China; 19949838599@163.com; 2 School of Electronic and Information Engineering/School of Integrated Circuits, Guangxi Normal University, Guilin 541004, China; 3 School of Chemistry and Chemical Engineering, Jishou University, Jishou 416000, China; 4 Institute of Physics, Bahauddin Zakariya University, Multan 66000, Pakistan; 5 Shandong Innovation Laboratory of Intelligent Computational Biology and Biosensing, Institute of Biophysics, Dezhou University, Dezhou 253023, China; 6 State Key Laboratory of Fabrication Technologies for Integrated Circuits, Institute of Microelectronics, Chinese Academy of Sciences, Beijing 100029, China; 7 University of Chinese Academy of Sciences, Beijing 100049, China

**Keywords:** terahertz, absorber, VO_2_, graphene, multi-mode tunability

## Abstract

Terahertz waves combine the dual characteristics of electronics and photonics and boast tremendous application potential in fields including nondestructive testing, telecommunications and biochemical sensing. Natural materials generally interact weakly with terahertz waves. In contrast, the reversible metal–insulator transition of VO_2_ and the tunable Fermi energy of graphene provide an effective route for developing dynamically reconfigurable terahertz devices. Existing VO_2_-graphene hybrid absorbers generally suffer from limitations such as single absorption mode, unsatisfactory absorption performance and complex multi-layer configurations. In this work, a reconfigurable five-layer terahertz metamaterial absorber supporting three operating modes is proposed. It consists of a patterned graphene top layer, an embedded patterned VO_2_ layer, and a bottom metallic reflector that suppresses terahertz transmission. The study is primarily conducted using the electromagnetic simulation software CST, and the physical mechanism behind the absorption peaks’ formation is elucidated via impedance matching theory and electric field energy distribution profiles. Simulation results demonstrate that the device supports three operating modes—dual narrowband, single narrowband and broadband—with peak absorptivity above 99% in each mode. The absorption spectral response can be dynamically modulated by regulating the Fermi energy level of graphene and modifying the material properties of VO_2_ via thermal regulation. Meanwhile, the proposed absorbing device sustains robust high-absorption performance across polarization orientation angles of 0–80° and incident angles of 0–50°. The dual-narrowband and single-narrowband modes are applicable to refractive index sensing, delivering a maximum sensitivity of 1750 GHz/RIU and a figure of merit (FOM) as high as 5.13. Finally, compared to existing similar devices, the proposed five-layer structure integrates multi-mode switching capability and high sensitivity, thus presenting promising application prospects. The fabrication of the patterned graphene and VO_2_ layers at different planes, however, still requires careful multilayer alignment and should be considered in practical implementation.

## 1. Introduction

Terahertz waves combine the dual characteristics of electronics and photonics, featuring low photon energy and no ionizing radiation [1]. This waveband also has excellent penetration capability, for example, it can penetrate non-metallic and non-polar materials such as clothing, plastics and paper, and shows significant characteristic absorption responses to water and various polar molecules [2]. In addition, terahertz waves have the advantages of broad bandwidth and high resolution [3,4]. These unique properties make the development of terahertz technology of immeasurable value, and demonstrate its great application potential in fields including high-speed communication, security shielding, non-destructive testing and biomedicine [5,6,7]. However, realizing the above applications requires efficient and flexible manipulation of terahertz waves, which is a key technical difficulty at present. Most natural materials have extremely weak response to terahertz waves and cannot directly achieve effective wave control, which greatly restricts the development of related technologies. This has prompted researchers to explore a new type of artificial electromagnetic material—metamaterials [8,9,10]. Metamaterials consist of subwavelength unit cells made from metals or dielectric materials. Through artificially designed structural patterns, they can produce unique electromagnetic behaviors unavailable in natural substances and realize the regulation of electromagnetic waves, providing a brand-new technical path to break through the bottleneck of terahertz wave manipulation [11,12]. Metamaterials can achieve a variety of attractive properties and impressive applications, such as negative refraction, wave focusing and electromagnetically induced transparency, which provide research ideas for the development of terahertz devices [13,14,15].

Terahertz metamaterial absorbers constitute a key branch in this research domain. Featuring subwavelength thickness, near-unity absorptivity and tunable electromagnetic parameters, they hold promising application potential in fields including perfect absorption and sensing devices [16,17,18]. In 2021, Aruna et al. designed an ultra-compact, high-sensitivity terahertz metamaterial biosensor [19]. At 4.87 THz, the sensor achieves an absorptivity reaching 99.9%, and the average sensitivity is 1936 GHz/RIU. However, it lacks dynamic tunability: once fabricated, its resonant frequency remains fixed, making it difficult to meet the demand for dynamic tunability in modern systems [20]. Driven by recent advances in materials science and micro/nanofabrication technologies, research on metamaterial absorbers has gradually shifted toward tunable and multifunctional designs [21]. Materials such as graphene [22,23], Dirac Semimetal [24] and vanadium dioxide (VO_2_) [25] exhibit excellent optical tunability and are extensively used to achieve dynamic regulation of absorption performance. The development of such novel absorbers not only opens up new avenues for the design and application of electromagnetic wave-absorbing materials, but also lays a solid foundation for technological innovation in fields including communications and national defense.

Vanadium dioxide (VO_2_) is a typical phase change material (PCM) characterized by its metal–insulator transition property [26]. Specifically, it undergoes a distinct reversible semiconductor-to-metal phase transition (Metal–Insulator Transition, MIT) around 341 K. Owing to this unique feature, VO_2_ has been utilized to fabricate tunable terahertz photonic devices, and extensive research has been carried out on its thermally controlled tunability. Upon phase transition, VO_2_ transforms into a tetragonal crystal structure (R phase) with metallic properties, and its electrical conductivity increases dramatically, varying between 20 S/m and 200,000 S/m [27]. Vanadium dioxide (VO_2_) is a typical phase-change material that undergoes a reversible insulator-to-metal transition near 341 K [28]. During this transition, its electrical conductivity changes by several orders of magnitude, from approximately 20 S/m in the insulating state to approximately 2 × 10^5^ S/m in the metallic state. This conductivity modulation provides an effective means of thermally tuning the electromagnetic response of terahertz devices.

Graphene is a two-dimensional conductive material with excellent carrier transport properties [29,30]. Its surface conductivity and resonant response can be continuously regulated by varying its Fermi level through electrostatic gating or chemical doping. Thus, graphene provides an electrically tunable mechanism that complements the thermally induced conductivity switching of VO_2_. Graphene, a unique carbon allotrope with a two-dimensional honeycomb lattice structure, exhibits excellent electron transport properties. Compared with conventional metallic materials like Au, Ag and Al, graphene supports surface plasmon resonance via the carriers’ direct transition within its band gap, and modulation of its Fermi level is achievable by means of chemical doping treatment or the application of gate voltage [31]. Farooq et al. reported an optically transparent terahertz metasurface based on graphene, which demonstrates wide-angle stability for biosensing applications to address these challenges [32]. Fosteris et al. proposed a design of an optical transparent, flexible, and adjustable microwave absorber that covers the X and Ku frequency bands. The absorber is based on a metasurface composed of a periodic array of graphene spiral resonators (GSRs) attached to an ultra-thin PET film placed on an ITO backed dielectric spacer [33]. Utilizing the phase transition behavior of vanadium dioxide, it realizes seamless switching between two contiguous broadband absorption bands. Nevertheless, the switching occurs exclusively between different broadband modes, with relatively single functionality.

Recent graphene-metasurface biosensors have demonstrated the potential of terahertz sensing for non-invasive cancer detection [34]. Graphene metasurface systems have also been applied to direct dopamine detection through terahertz surface plasmon resonance and machine-learning-assisted analysis [35]. In addition, graphene and plasmonic nanostructures have been integrated with terahertz metasurfaces for high-sensitivity glucose detection with machine-learning optimization [36]. These studies demonstrate the broad application potential of graphene-based terahertz biosensors, whereas the present work focuses on integrating dynamically switchable multi-mode absorption with refractive-index sensing in a graphene–VO_2_ hybrid structure.

In summary, despite the progress made in narrowband and broadband absorption as well as preliminary tunability, current terahertz perfect absorbers have yet to achieve multi-mode tunable functionality. Specifically, existing devices generally present three main limitations. First, they have single functional modes, with most designs only supporting broadband-to-broadband switching. Second, the fabrication complexity of tunable absorbers is determined not only by the number of stacked layers, but also by the number and arrangement of active materials, as well as the patterning and interlayer alignment requirements. In particular, structures incorporating patterned active materials on different planes generally require multiple fabrication steps and precise alignment. Therefore, although some reported devices employ six or more layers, a five-layer configuration containing separately patterned graphene and VO_2_ layers may still involve considerable fabrication complexity. Third, although refractive-index sensing is a common response of metamaterial absorbers with localized resonant fields, the sensing characteristics of different switchable absorption modes have not always been quantitatively evaluated. Therefore, it remains of interest to examine the refractive-index response of narrowband modes in multifunctional and dynamically tunable absorbers. Accordingly, enabling dynamic reconfiguration of absorption modes (broadband/narrowband/multi-frequency) and operating states (absorption/transmission/reflection), while simultaneously integrating high-sensitivity sensing and detection functions, has become a key scientific challenge. Although the proposed device employs a five-layer configuration with patterned graphene and VO_2_ active layers, its practical fabrication requires careful multilayer patterning and alignment. Therefore, this work focuses on realizing multifunctional absorption and sensing performance within a compact five-layer configuration.

## 2. Structural Model and Basic Theory

**Figure 1:**
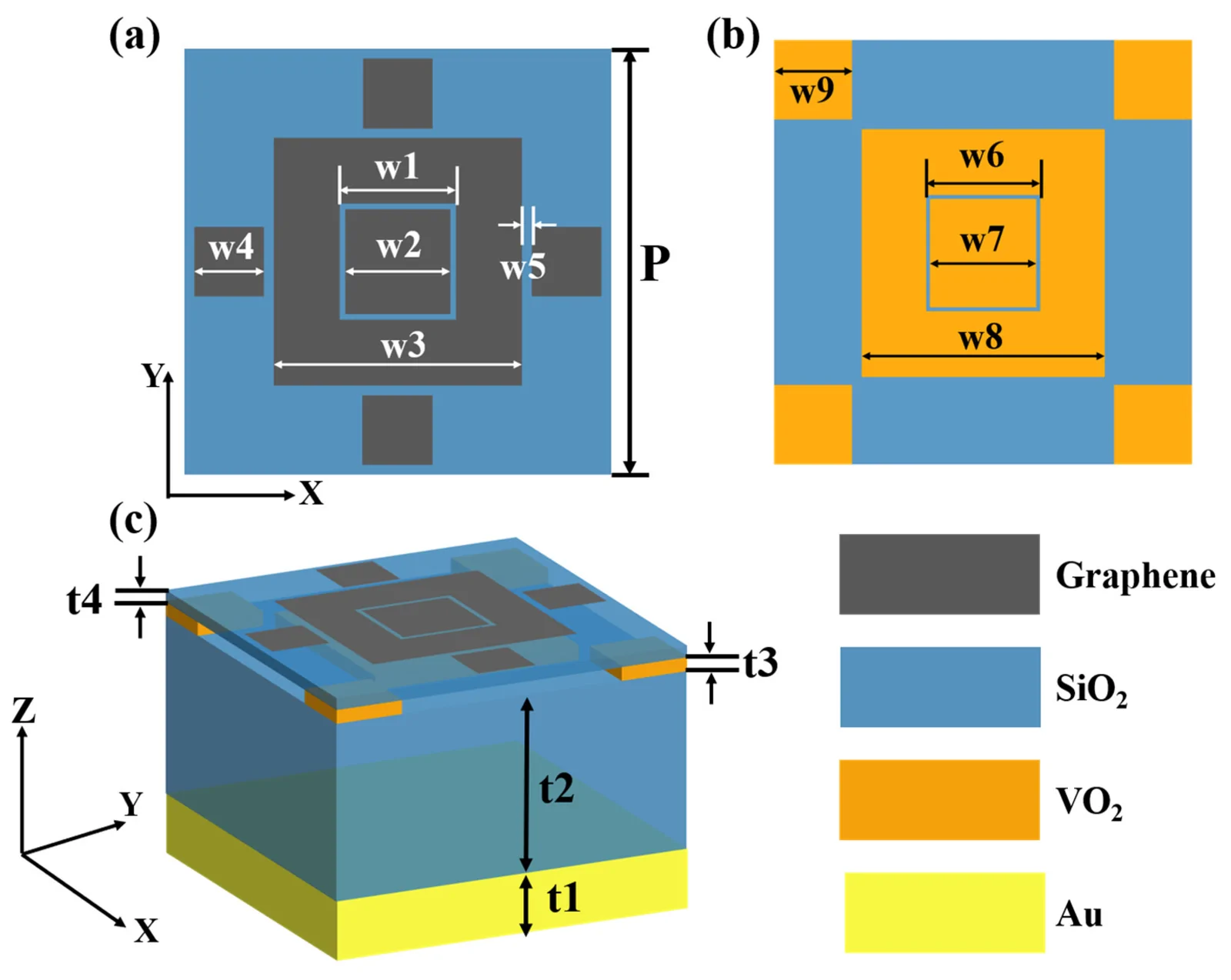
(**a**) Top view in the X–Y plane of the top-layer graphene of the absorber. (**b**) Top view in the X–Y plane of the vanadium dioxide layer of the absorber. (**c**) Schematic diagram of the three-dimensional structure of the absorber.

The proposed tri-functional tunable terahertz metamaterial absorber is schematically illustrated in Figure 1. The bottom-most layer uses gold substrate with a thickness defined as t1 = 5 μm, whose thickness is much larger than the skin depth, capable of effectively obstructing the propagation of electromagnetic waves. Above the gold substrate lies a silicon dioxide layer with a relative permittivity (εr) of 2.13 and a thickness t2 of 10.7 μm. The third layer consists of patterned vanadium dioxide (VO_2_), whose geometric pattern is shown in Figure 1b: a square ring at the center with four small squares attached at the four corners. Table 1 and Table 2 provide the detailed structural parameters, and the VO_2_ layer t3’s thickness is 0.1 μm. An additional silicon dioxide layer is deposited on top of the VO_2_ layer, with a thickness t4 of 1.2 μm. The uppermost layer consists of patterned graphene with a 1 nm thickness. Its pattern, as shown in Figure 1a, also has a square ring at the center, with small squares arranged around its periphery. A summary of the relevant parameters is presented in Table 1 and Table 2.

Vanadium dioxide (VO_2_) has attracted extensive research attention owing to its distinctive physical properties. When subjected to temperature variations within a specific range, VO_2_ undergoes an insulator-to-metal transition (IMT) accompanied by lattice structural changes [37]. Its electrical conductivity changes dramatically at different temperatures: within a certain interval, it can increase from approximately 20 S/m to a maximum on the order of 2 × 10^5^ S/m. At room temperature, its relatively high electrical conductivity is conducive to the development of optoelectronic devices.

Within the terahertz (THz) frequency range, the dielectric permittivity of vanadium dioxide can be characterized using the Drude model [38]:

(1) $$E_{{VO}_{2}}=\varepsilon_{\infty ,{VO}_{2}}-\frac{\omega_{p,{VO}_{2}}^{2}}{\omega_{p,{VO}_{2}}^{2}+i\omega \gamma_{{VO}_{2}}}$$

The symbol $\varepsilon_{\infty ,{VO}_{2}}$ indicates the infinite-frequency permittivity of VO_2_ at a magnitude of 12, while $\gamma_{{VO}_{2}}$ signifies collision frequency with a value of 5.75 × 10^13^ rad/s. Meanwhile, ω stands for the angular frequency of incident electromagnetic radiation, and $\omega_{p{,VO}_{2}}\left(\sigma \right)$ is used to express plasma frequency.

**Figure 2:**
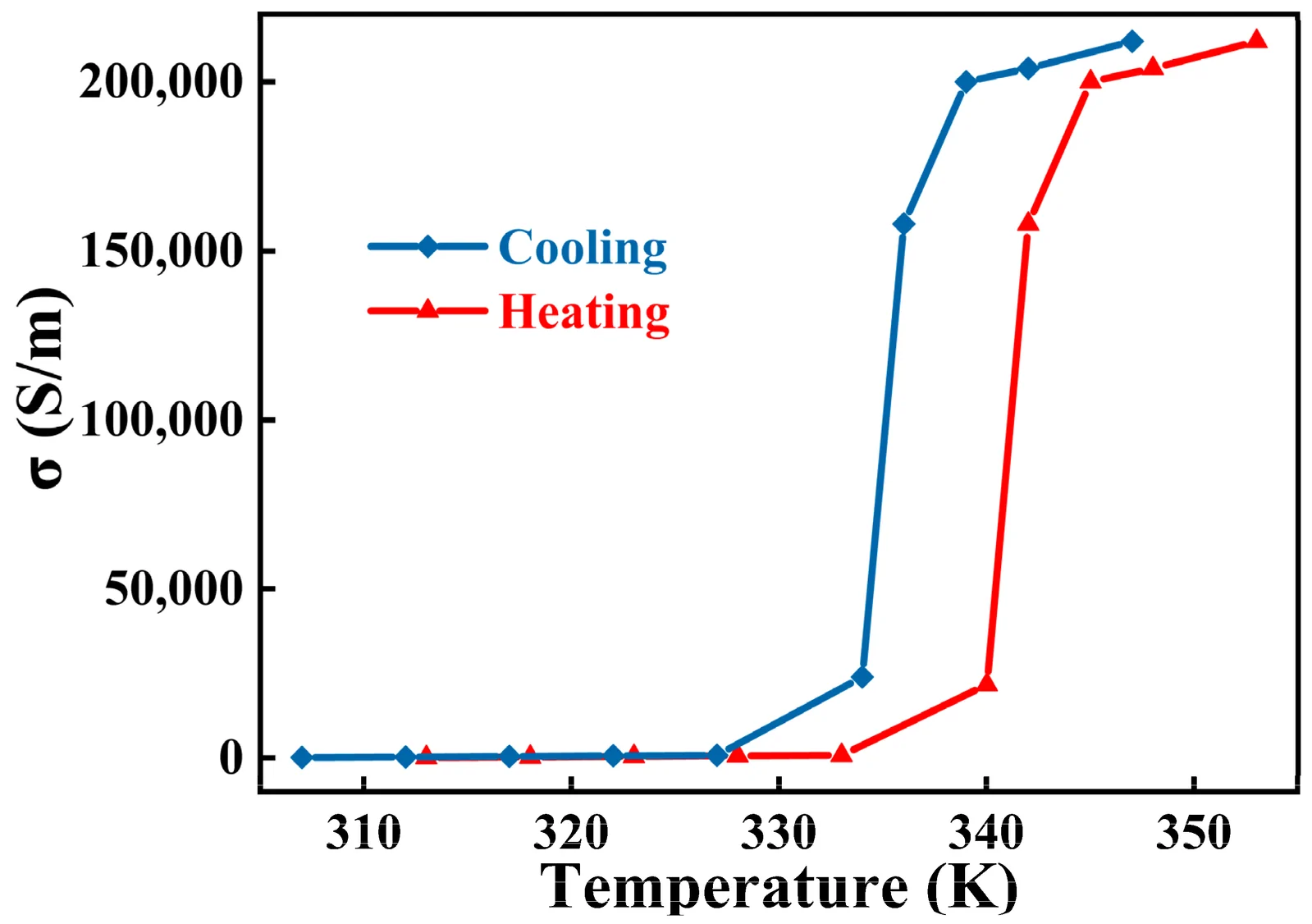
Curve of electrical conductivity of vanadium dioxide versus ambient temperature.

$\omega_{p{,VO}_{2}}\left(\sigma \right)$ is the conductivity-dependent plasma frequency, which can be approximately expressed as [39]

(2) $$\omega_{p,{VO}_{2}}^{2}\left(\sigma \right)=\frac{\sigma }{\sigma_{0}}\omega_{p,{VO}_{2}}^{2}\left(\sigma_{0}\right)$$

where $\omega_{p,{VO}_{2}}\left(\sigma_{0}\right)=1.4\times 10^{15}rad/s$ and $\sigma_{0}=3\times 10^{5}$ S/m. The electrical conductivity σ of VO_2_ varies with its phase transition. As temperature changes, VO_2_ can be reversibly switched between its insulating state and metallic state, and the variation in its electrical conductivity is synchronized with the temperature variation [40]. The relationship between the electrical conductivity of VO_2_ and the ambient temperature is shown in Figure 2. The temperature-dependent change in VO_2_ electrical conductivity mainly arises from the temperature-induced variation in its dielectric constant.

The surface conductivity of graphene is an important parameter characterizing its metallic properties [41], and it also serves as the foundation for designing actively tunable graphene metamaterial absorbers. To describe the electromagnetic response of graphene under electromagnetic fields, the classic Kubo model is adopted to express the conductivity of graphene [42], which is given as follows:

(3) $$\Sigma =\sigma_{intra}+\sigma_{inter}$$

In the above formula, σintra and σinter are expressed as follows, respectively:

(4) $$\sigma_{intra}=\frac{ie^{2}k_{B}T}{\pi \hbar^{2}\left(\omega +i\tau^{-1}\right)}\left[\frac{E_{F}}{K_{B}T}+2\ln\left(e^{-\frac{E_{F}}{k_{B}T}}+1\right)\right]$$

(5) $$\sigma_{inter}=\frac{ie^{2}}{4\pi \hbar^{2}}\ln\left[\frac{2\left|E_{F}\right|-\hbar \left(\omega +i\tau^{-1}\right)}{2\left|E_{F}\right|+\hbar \left(\omega +i\tau^{-1}\right)}\right]$$

In formulas, the electrical conductivity is denoted by σ, the elementary charge is represented by *e*, the imaginary unit is given by *i*, the Boltzmann constant is marked as $k_{B}$, the reduced Planck constant is denoted by ℏ, the thermodynamic temperature is indicated by *T*, the Fermi level of graphene is defined as *E_F_*, the relaxation time of graphene is characterized by τ, and the angular frequency of electromagnetic excitation is expressed by ω.

The total conductivity of graphene metamaterials arises from two components-intraband conductivity σintra and interband conductivity σinter-which exert different degrees of influence. The frequency band explored in this paper falls into the terahertz range. When EF≫ℏω is satisfied, intraband transitions account for the majority of the conductivity, whereas the contribution of interband transitions can be omitted. Drawing on the Drude model, graphene’s conductivity can be simplified as the expression below [43]:

(6) $$\sigma =\frac{e^{2}E_{F}}{\pi \hbar^{2}}\frac{i}{\omega +i\tau^{-1}}$$

(7) $$\varepsilon =1+\frac{i\sigma }{\omega \varepsilon_{0}}$$

where ε is the dielectric constant of graphene, and $\varepsilon_{0}$ denotes the permittivity of free space;

The dielectric function of gold adopted in this work is described by the classical Drude model for lossy gold [44]:

(8) $$E_{Au}=\varepsilon_{\infty ,Au}-\frac{\omega_{p,Au}^{2}}{\omega_{p,Au}^{2}+i\omega \gamma_{Au}}$$

where $\varepsilon_{\infty ,Au}=9.1$ denotes the high-frequency limit dielectric constant. The plasma frequency $\omega_{p,Au}$ = 1.3659 × 10^16^ rad/s and collision frequency $\gamma_{Au}=$ 1.0318 × 10^14^ rad/s are adopted in this work.

The absorptivity is calculated by the formula A = 1 – R − T = 1 − S_11_|^2^ − |S_21_|^2^, where R and T represent the reflectivity and transmittance of the absorber, respectively [45,46,47]. R = |S_11_|^2^ and T = |S_21_|^2^ are derived from S-parameters [48]. Since the bottom gold reflective layer fully obstructs electromagnetic radiation, the transmittance equals zero (T = 0), which demonstrates that the level of absorption is entirely dictated by the reflection intensity [49].

The absorber was simulated in CST using periodic boundary conditions in the x and y directions and Floquet-port excitation along the z direction. Gold, SiO_2_, and VO_2_ were modeled as three-dimensional materials, while graphene was treated as a 1 nm conductive sheet described by its frequency-dependent Kubo surface conductivity [50]. An adaptive mesh refinement procedure was applied, with particular refinement around the graphene and VO_2_ patterns, narrow gaps, and material interfaces. The results were considered converged when further mesh refinement caused negligible changes in the resonance frequencies and absorptivity spectra [51]. The VO_2_ response was determined by its conductivity-dependent dispersive model, and the graphene model was updated for each specified Fermi energy.

## 3. Results and Analysis

In this work, we realize the design of multi-mode tunable absorption performance for a novel composite material by regulating the Fermi level of graphene and the electronic structure of vanadium dioxide (VO_2_), as shown in Table 3. In the simulations, the VO_2_ conductivity is set to 20 S/m to represent the insulating state and to 2 × 10^5^ S/m to represent the metallic state. These two conductivity values are used to evaluate the effect of the VO_2_ state on the absorption response.

To maintain consistency between the material model and the operating conditions, the conductivity values listed in Table 3 are treated as effective conductivities obtained from the temperature-dependent curve in Figure 2. The value of 20 S/m corresponds to the insulating state at approximately room temperature and is used for Mode I. The intermediate value of 302.5 S/m used in Mode III represents a partially transformed VO_2_ state located within the transition region. It should therefore be associated with an intermediate temperature close to the onset or middle of the metal–insulator transition, rather than with a fully insulating or fully metallic state. The high conductivity of $2\times 10^{5}$ S/m used in Mode II corresponds to the metallic state after the transition is completed.

The exact temperatures associated with 302.5 S/m and $2\times 10^{5}$ S/m should be determined from the calibrated conductivity–temperature curve of the fabricated VO_2_ film, because the transition temperature and transition width depend on film thickness, crystallinity, strain, and thermal boundary conditions. In the present simulations, conductivity is used as the primary material-control parameter, while temperature is the external variable that produces this conductivity change. Thus, the three modes are selected by stabilizing VO_2_ at the temperature required to obtain the corresponding effective conductivity.

The heating and cooling curves in Figure 2 should also be interpreted as hysteretic transition paths. During heating, VO_2_ changes from the insulating state toward the metallic state, whereas during cooling the reverse transition occurs at a different temperature. Consequently, the same conductivity may be reached at slightly different temperatures depending on the previous thermal history. This hysteresis does not invalidate the three-mode operation, but it implies that the temperature values for switching should be calibrated separately for heating and cooling cycles. In practice, a feedback temperature controller should be used to stabilize the VO_2_ film at the conductivity required for each mode.

**Figure 3:**
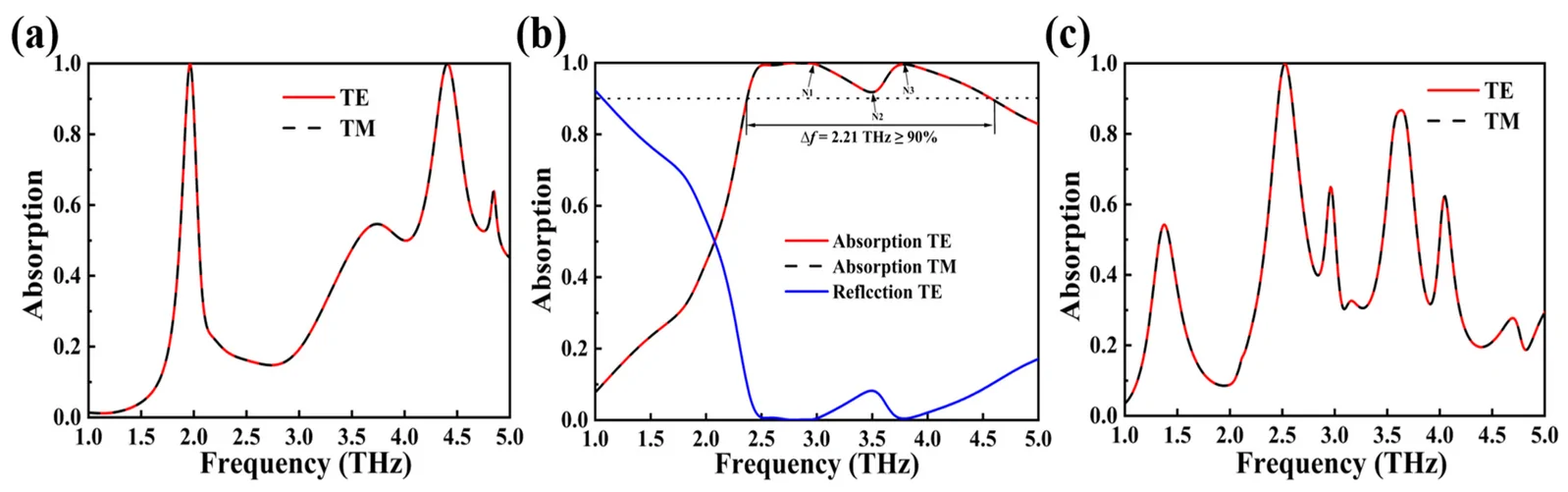
(**a**) Absorption spectrum of absorption Mode I. (**b**) Absorption spectrum of absorption Mode II. (**c**) Absorption spectrum of absorption Mode III.

The relaxation time of graphene is uniformly set to 1 ps across all three modes. To obtain the dual-frequency narrowband absorber in Mode I, the graphene’s Fermi level is set to 0.73 eV, while the VO_2_’s electrical conductivity is fixed at 20 S/m. At room temperature, the absorber ultimately achieves an absorptivity of 99.99% at 1.96 THz and 99.91% at 4.41 THz, with an average absorptivity of 99.95%. To realize the broadband absorber in Mode II, the graphene Fermi level is set to 0.3 eV and the VO_2_ conductivity is set to 2 × 10^5^ S/m, corresponding to the metallic-state parameter used in the simulation. The average absorptivity hits 96.62% across the frequency interval spanning 2.37–4.58 THz, yielding an effective bandwidth of 2.21 THz. Such absorption characteristic originates from the cooperative coupling effect between VO_2_ and graphene. For realizing the single-frequency narrowband absorption response in Mode III, graphene is configured with a Fermi level of 0.3 eV, while VO_2_ is assigned an electrical conductivity value of 302.5 S/m. In this configuration, VO_2_ stays within its insulating state, and the designed absorber realizes near-perfect absorption with an absorption rate of 99.85% at 2.53 THz. The absorption spectra for Modes I, II, and III are shown in Figure 3a, 3b, and 3c, respectively. The three operating modes can be realized by independently tuning the graphene Fermi energy and the VO_2_ conductivity. The patterned graphene may be connected to metal contacts located outside the active resonant region and controlled by electrostatic gating. The upper SiO_2_ layer can serve as the gate dielectric with an additional insulated top gate, while the bottom metal can alternatively be used as a common back gate with suitable electrical isolation. Thus, the simulated Fermi levels of 0.73 eV for Mode I and 0.3 eV for Modes II and III can be obtained by selecting appropriate gate voltages and dielectric parameters. The VO_2_ conductivity can be controlled using a microheater or a temperature-controlled substrate. At low temperature, VO_2_ remains insulating, corresponding approximately to Mode I. Heating through the metal–insulator transition produces the high-conductivity state required for Mode II, whereas an intermediate temperature within the transition region can provide the effective conductivity used for Mode III. Since the transition depends on film thickness, crystallinity, hysteresis, and thermal boundary conditions, the conductivity values in Table 3 should be calibrated for the fabricated VO_2_ film. The electrodes, bias lines, heater, and thermal-spreading layers may introduce additional capacitance, inductance, dielectric loss, or temperature nonuniformity, thereby shifting the resonances or reducing the absorption. These components should therefore be placed outside the high-field region whenever possible, and their effects should be considered in future full-wave electrothermal simulations.

**Figure 4:**
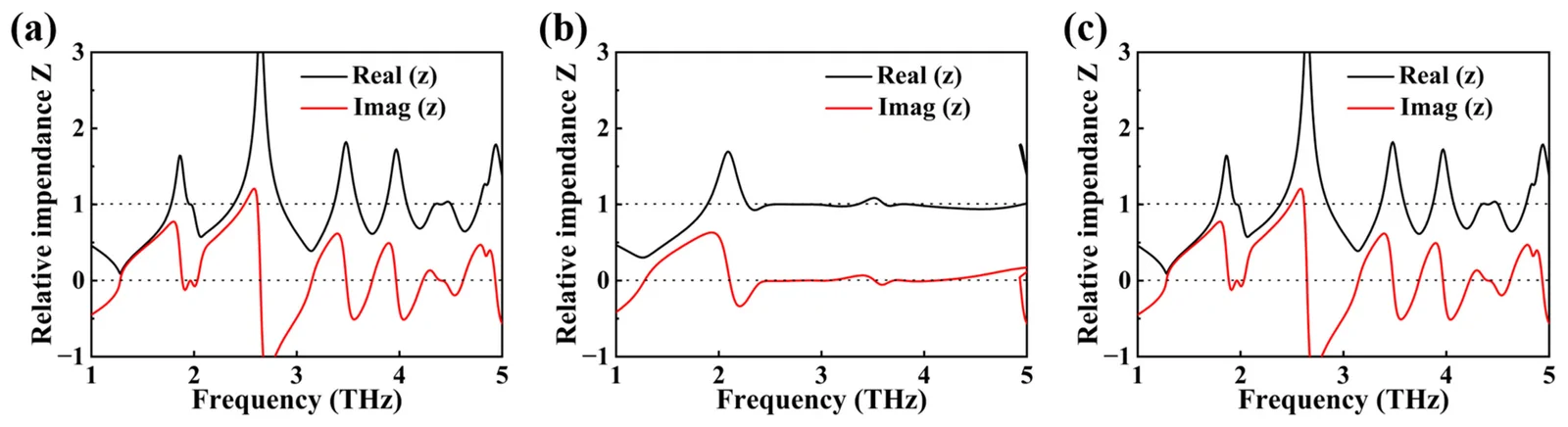
(**a**) Impedance diagram of Mode I. (**b**) Impedance diagram of Mode II. (**c**) Impedance diagram of Mode III.

Figure 4 shows the impedance matching spectra of the three absorption modes of the absorber designed in this study. As discussed in the second section of this study, impedance matching between the material and incident electromagnetic waves enables electromagnetic energy to penetrate the bulk of the material, which in turn weakens reflection and amplifies absorption efficiency [52,53]. Under this condition, the real part of the complex impedance is approximately unity and its imaginary part remains nearly zero, allowing electromagnetic waves to enter the material and be maximally absorbed. To verify whether the proposed absorber meets the impedance matching condition, we analyzed the impedance matching performance of the three absorption modes. The expression for the effective impedance $z_{eff}$ [54,55] is

(9) $$z_{\mathrm{eff}}=\pm \sqrt{\frac{{\left(1+S_{11}\right)}^{2}-S_{21}^{2}}{{\left(1-S_{11}\right)}^{2}-S_{21}^{2}}}$$

For absorption Mode I, at the absorption peak of 1.96 THz, the real part approaches 1 and the imaginary part approaches 0, indicating that impedance matching is achieved at this frequency. For the absorption peak at 4.41 THz, the real part and the imaginary part approaches 1 and 0, respectively, which also satisfies impedance matching. However, the fitting quality of the imaginary part at 4.41 THz is inferior to that at 1.96 THz, resulting in a relatively lower absorptivity. For absorption Mode II, as shown in Figure 4b, for relative impedance, its real value approaches unity and the imaginary value evolves toward zero across the 2.37–4.58 THz band. Nevertheless, a deviation occurs near 3.50 THz with poor impedance matching performance [56], leading to a lower absorptivity of 91.78% at this frequency point. For absorption Mode III, as shown in Figure 4c, the real part of relative impedance converges to unity, with the imaginary part trending toward zero at 2.53 THz, corresponding to an absorptivity of 99.85%. It can be observed from Figure 3c that there is an additional absorption peak at 3.64 THz in Mode III, but its absorptivity is merely 86.71%. The unsatisfactory relative impedance fitting at this frequency is responsible for the low absorptivity, which also indicates that part of the electromagnetic wave is reflected [57].

**Figure 5:**
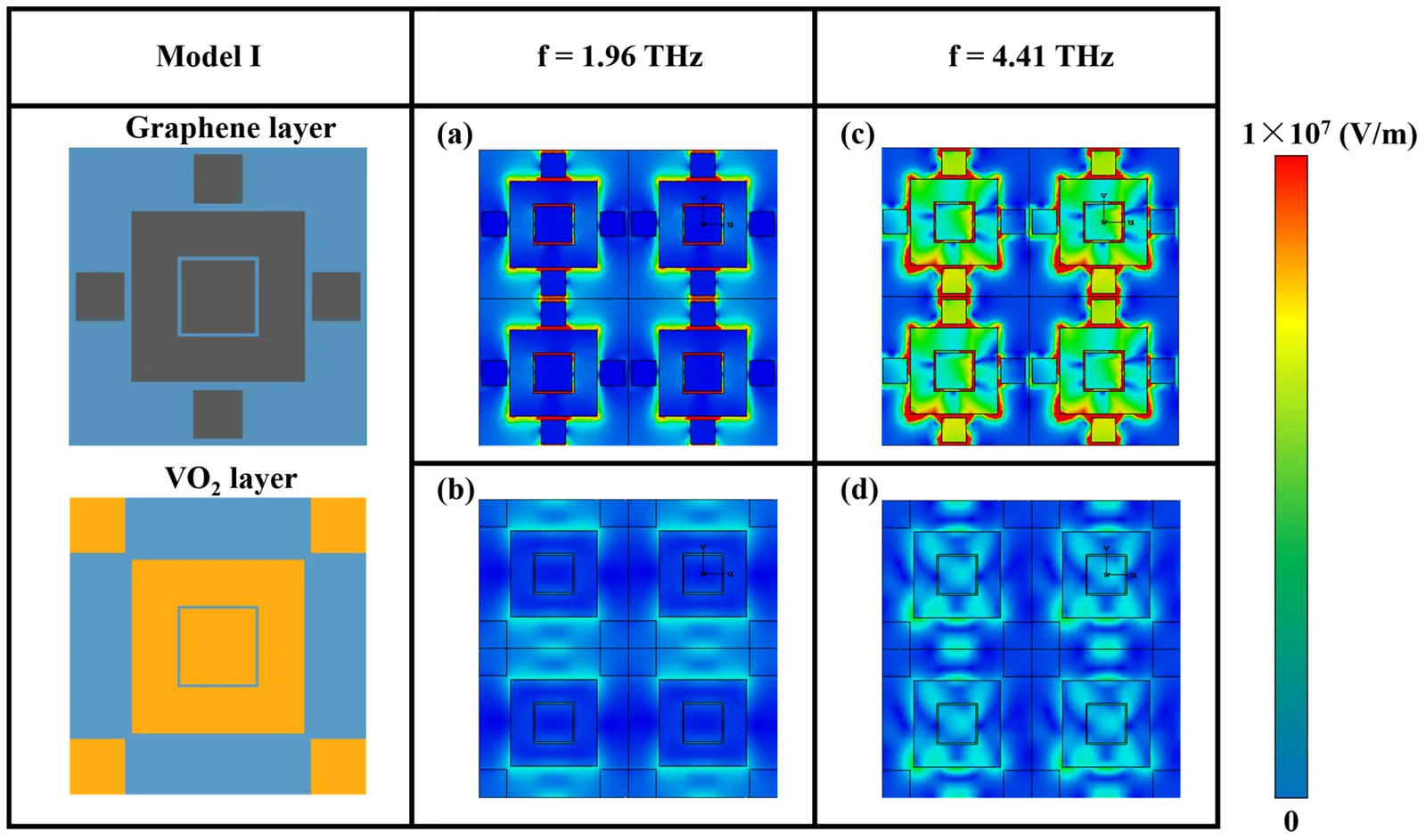
(**a**) Electric field energy distribution of the graphene layer for Mode I at 1.96 THz. (**b**) Electric field energy distribution of the graphene layer for Mode I at 4.41 THz. (**c**) Electric field energy distribution of the VO_2_ layer for Mode I at 1.96 THz. (**d**) Electric field energy distribution of the VO_2_ layer for Mode I at 4.41 THz.

**Figure 6:**
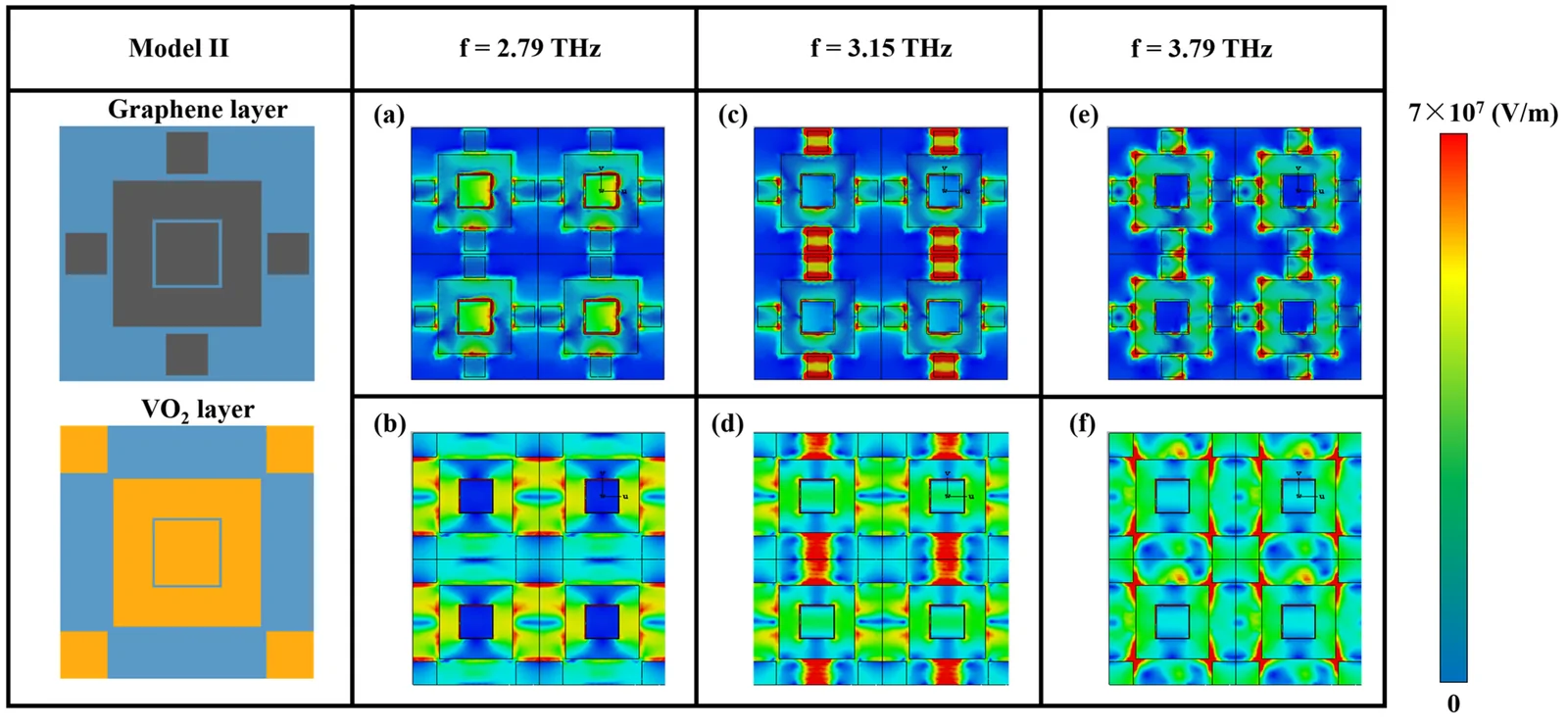
(**a**) Electric field energy distribution of the graphene layer for Mode II at 2.79 THz. (**b**) Electric field energy distribution of the graphene layer for Mode II at 3.15 THz. (**c**) Electric field energy distribution of the graphene layer for Mode II at 3.79 THz. (**d**) Electric field energy distribution of the VO_2_ layer for Mode II at 2.79 THz. (**e**) Electric field energy distribution of the VO_2_ layer for Mode II at 3.15 THz. (**f**) Electric field energy distribution of the VO_2_ layer for Mode II at 3.79 THz.

**Figure 7:**
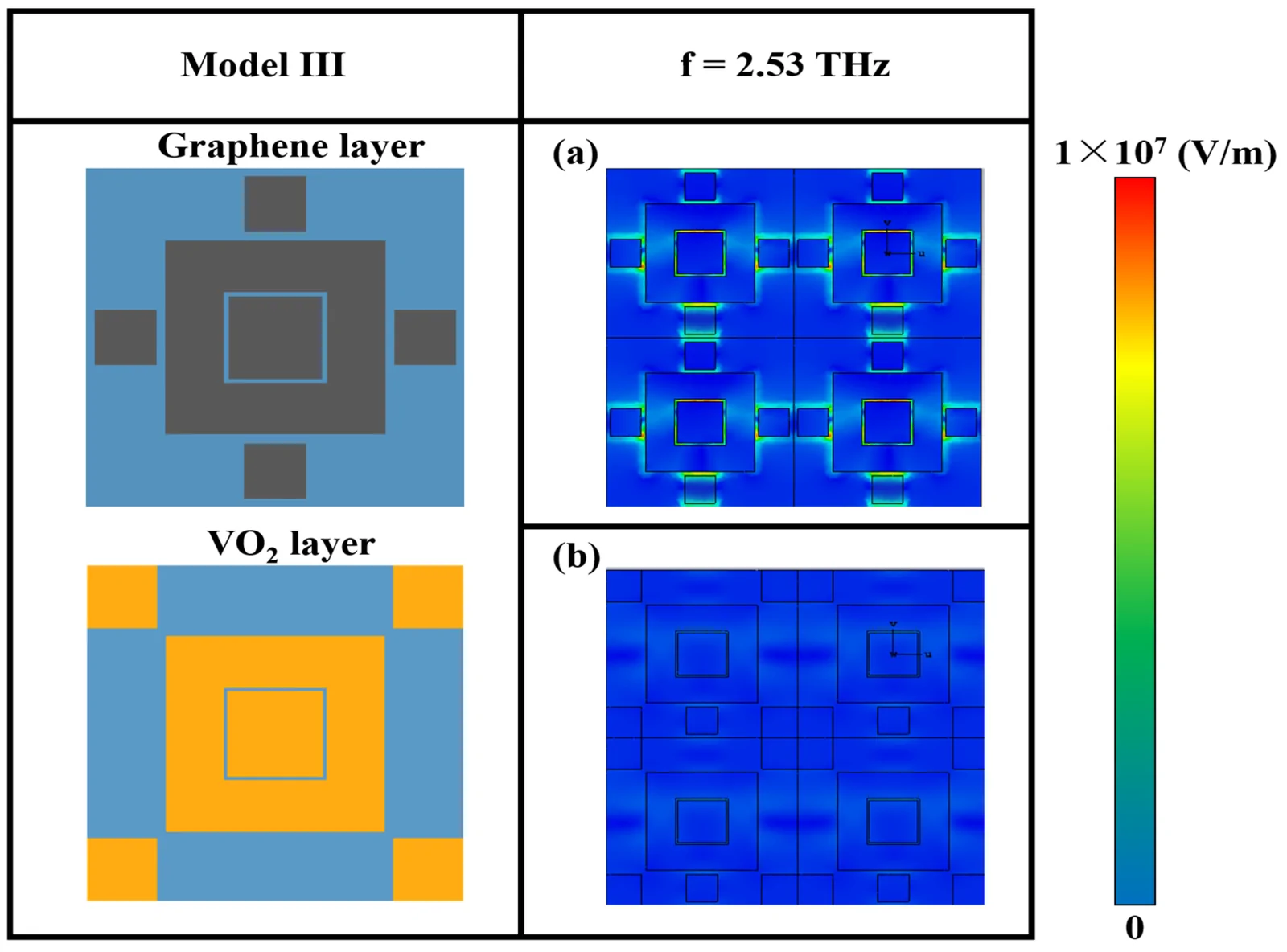
(**a**) Electric field energy distribution of the graphene layer for Mode III at 2.53 THz. (**b**) Electric field energy distribution of the VO_2_ layer for Mode III at 2.53 THz.

To better clarify the absorption behaviors, we analyze the electric field distributions at discrete frequency values, as illustrated in Figure 5, Figure 6 and Figure 7. In Mode I, the electric field predominantly resides within the graphene layer domain, due to pronounced surface plasmon resonance occurring here [58,59]. The highly concentrated local electric field profile within this zone pushes the energy absorption efficiency of the absorber against incident electromagnetic radiation up to its maximum value [60]. In contrast, barely any electric field energy exists in the vanadium dioxide (VO_2_) layer. This is because VO_2_ in this mode stays at room temperature with an electrical conductivity of 20 S/m, presenting an insulating phase. Furthermore, VO_2_ is a thin insulating material interposed between two silica films, constructing a comparatively homogeneous dielectric background alongside the adjacent SiO_2_ layers, resulting in the absence of obvious fluctuation or accumulation of the electric field within this region [61]. By comparison, the top graphene layer features ultrahigh electron mobility and excellent electrical conductivity, which confines the electric field to the vicinity of the surface, boosting localized field enhancement in this domain [62]. The above phenomena indicate that the graphene layer plays a dominant role in Mode I. For the absorption peak that at 1.96 THz, the corresponding strong electric field is mainly distributed in the graphene layer’s square gaps, while only a weak electric field is detected in the VO_2_ layer. For the absorption peak at 4.41 THz, compared to the case at 1.96 THz, the graphene layer inside the absorber possesses a higher level of energy. However, as noted in the impedance matching analysis, the fitting performance of its imaginary part is unsatisfactory. Therefore, although a strong electric field can be excited, part of the energy is reflected back to free space [63]. As illustrated in Figure 6 under Mode II configuration, electric field energy predominantly localizes at the square frame regions of the VO_2_ film, whereas the electric field strength inside the graphene layer is relatively feeble. This indicates that the generation of Mode II is primarily attributed to the VO_2_ layer: when VO_2_ enters the metallic state, its electrical conductivity is significantly enhanced, which can increase the absorption of incident electromagnetic waves; whereas when the Fermi level of graphene is low, the internal free carrier concentration decreases, resulting in a weakened response to electromagnetic waves. For the electric field at 2.79 THz in Mode II, we find that electromagnetic energy is mainly concentrated in the horizontal direction of the VO_2_ layer, while the graphene layer shows a tendency of local electric field enhancement in a small portion. This demonstrates that the high absorption at 2.79 THz is mainly driven by the VO_2_ layer. According to the electric field energy distribution corresponding to 3.15 THz in Mode II, strong electric fields exist in both the graphene layer and the VO_2_ layer at this frequency, and both are concentrated in the vertical structures. Nevertheless, the main electric field energy in the VO_2_ layer is not distributed in the patterned VO_2_. Combined with the previous impedance analysis, this arises from the poor fitting at this position. Accordingly, the structure acts as an efficient inductor-capacitor (LC) resonator, localizing the energy inside. Coupled with the mismatch between the equivalent impedance of the absorber and the free space impedance, most of the energy is reflected back to free space, leading to poor absorption performance [64,65]. For Mode III, the field distribution at 2.53 THz indicates that the absorption is primarily associated with the localized field enhancement in the graphene gaps, with a weaker contribution from the VO_2_ layer.

Finally, we also discuss the electric field distribution at the special resonance point of 2.53 THz in Mode III, as shown in Figure 7. The strong electric field at 2.53 THz in Mode III is mainly distributed at the gaps between graphene layers, but its intensity is lower than that in Mode I and Mode II, while the low-intensity electric field is dispersed at the boundaries of adjacent unit structures in the VO_2_ layer. The absorption effect at this point is mainly attributed to the graphene layer. It can be seen from the figure that the overall multilayer structure functions as a resonant system, and interlayer coupling effects emerge across different material films, which consequently produce distinct absorption properties [66,67].

To further verify the origin of each absorption peak, layer-resolved power-loss and surface-current distributions should be analyzed at the resonance frequencies. The integrated loss in the graphene and VO_2_ layers can distinguish graphene-dominated, VO_2_-dominated, and cooperative-coupling resonances [68]. In particular, simultaneous strong loss contributions from graphene and VO_2_ would provide quantitative evidence for the broadband coupling mechanism of Mode II. Therefore, the field distributions and impedance-matching results should be interpreted together with current and loss analyses.

**Figure 8:**
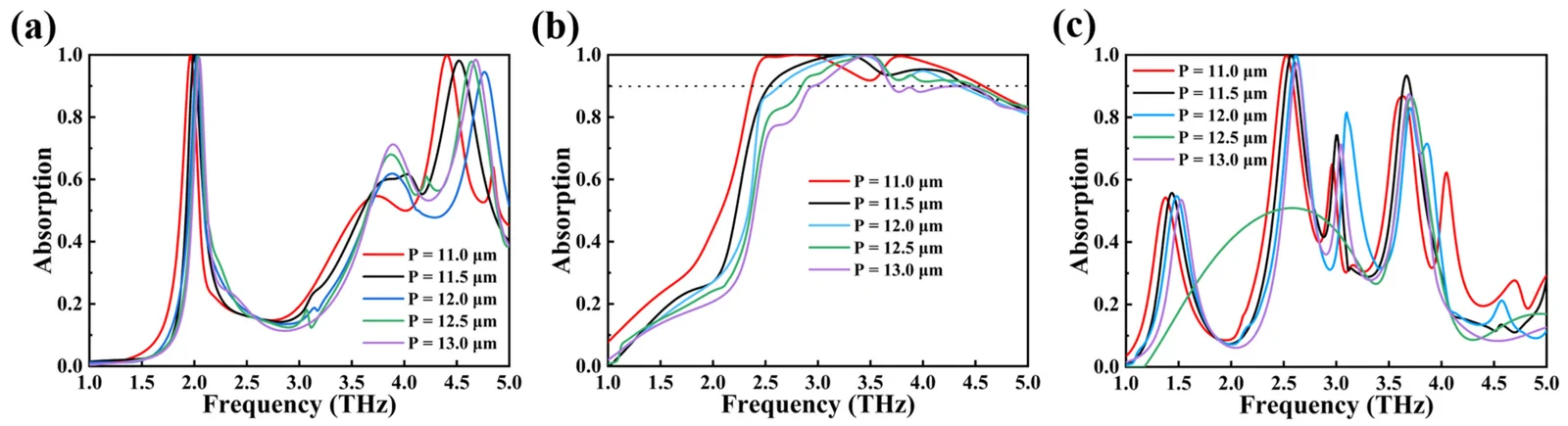
(**a**) Absorption spectra of Mode I at different periods. (**b**) Absorption spectra of Mode II at different periods. (**c**) Mode III’s absorption spectra at different periods.

Additionally, we investigate the effects of different structural parameters on the absorption performance, as shown Figure 8. For Mode I, with an increase in the period from 11.0 μm to 13.0 μm, the absorptivity of the absorber at 1.96 THz shows no significant change, while the absorption peak’s absorptivity at 4.41 THz decreases markedly and exhibits a blue-shift trend. For Mode II, as we adjust the period from 11.0 μm up to 13.0 μm, its absorption spectrum deteriorates significantly. For instance, at a period of 11.5 μm, the corresponding absorption bandwidth narrows and the absorptivity also degrades.

As the period P increases, the frequency corresponding to 90% absorptivity at the low-frequency band tends to blue-shift, while the frequency corresponding to 90% absorptivity at the high-frequency band remains nearly unchanged, leading to a gradual reduction in absorption bandwidth. For Mode III, although an absorption peak with over 90% absorptivity emerges at 3.67 THz when the period is 11.5 μm, this parameter setting will significantly degrade the performance of the first two modes. By comparing the absorption performance of the device under different structural parameters, it can be observed that for Mode I and Mode II, the absorption performance is superior when the structural dimension is set to 11 μm compared with other values. For Mode III, adjusting the parameter to 11.5 μm can yield an absorption peak exceeding 90% at 3.67 THz with prominent absorption performance. Nevertheless, this adjustment will notably impair the performance of Mode I and Mode II, resulting in a substantial decline in their absorption capacity. This work aims to design a structure that delivers favorable absorption performance across multiple modes, rather than focusing on the performance of a single mode [69]. Therefore, after comprehensively balancing the performance of the three modes, 11 μm is finally selected as the optimal structural parameter in this chapter. In addition, the above analysis demonstrates that different structural parameters exert distinct tuning effects on different modes. Accordingly, in practical applications, if a specific scenario has higher requirements for the performance of a certain mode, the structural parameters can be adjusted following this rule to satisfy the demands of various applications [70,71]. This also provides a reference for the flexible practical deployment of the device structure proposed in this chapter.

**Figure 9:**
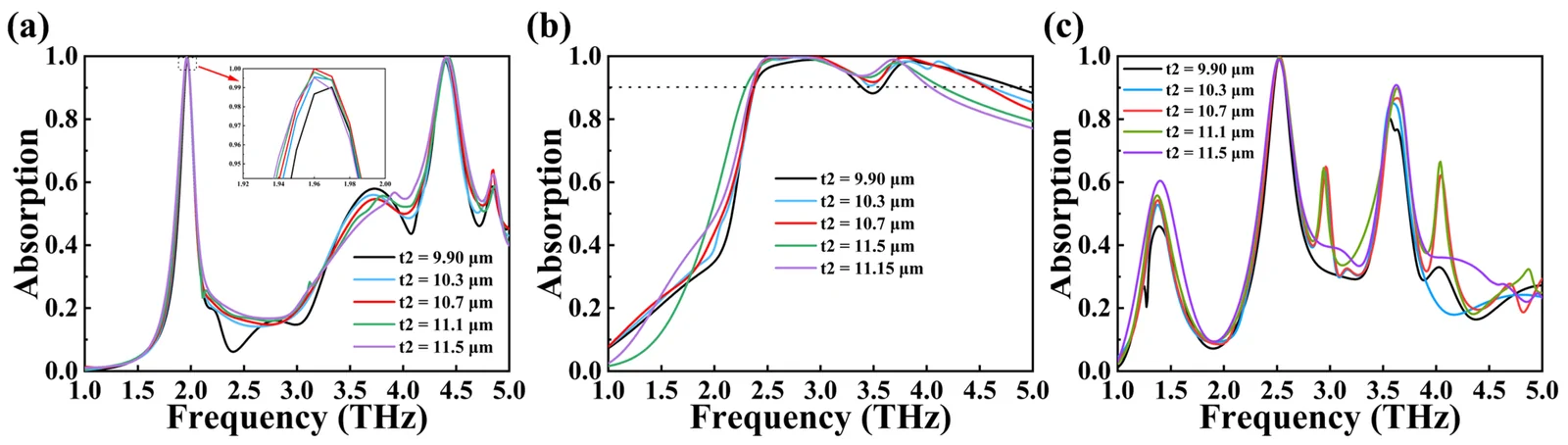
(**a**) Absorption spectra of Mode I with different t2 thicknesses. (**b**) Absorption spectra of Mode II with different t2 thicknesses. (**c**) Absorption spectra of Mode III with different t2 thicknesses.

Subsequently, in this paper, the influence of different thickness values of the SiO_2_ spacer layer t2 on the absorption characteristics is systematically analyzed. It can be seen from Figure 9, varying the value of t2 exerts negligible influence on the absorption spectrum of Mode I, and the two absorption peaks still maintain ultrahigh perfect absorption above 99%, indicating that the absorption response of Mode I is relatively insensitive to variations in the SiO_2_ spacer-layer thickness within the investigated range. For Mode II, the absorptivity drops below 90% when t2 = 9.9 μm; the average absorptivity reaches 96.25% at t2 = 10.3 μm; when t2 is less than 10.7 μm, the absorption bandwidth decreases gradually with the increase in t2. In summary, t2 = 10.7 μm is selected for structural design in this work. For Mode III, similar to Mode I, adjusting the value of t2 barely affects its absorption peaks. Overall, variations in the SiO_2_ spacer-layer thickness within the investigated range produce only minor changes in the absorption spectra, indicating that the absorption response is relatively stable with respect to t2. The influence of other geometric parameters, particularly the sub-micron patterned features, may require separate consideration because these parameters directly affect the local electromagnetic coupling and resonance conditions.

The tolerance analysis of the SiO_2_ spacer alone cannot fully represent the fabrication robustness of the absorber. Since Figure 8 shows that the response is sensitive to the period, realistic deviations in the patterned graphene and VO_2_ layers should also be considered. Variations in linewidth, feature size, gap width, and the alignment between the two patterned layers may change the effective capacitance, inductance, and near-field coupling, resulting in resonance shifts or reduced absorption. Therefore, the fabrication tolerance should be evaluated by varying the critical dimensions of both patterns around their nominal values and monitoring the resonance frequency, peak absorptivity, and bandwidth in all three modes [72,73]. The present conclusion should consequently be limited to the tolerance of the SiO_2_ spacer thickness and should not be extended to the complete absorber before the patterned dimensions and interlayer alignment are systematically analyzed.

**Figure 10:**
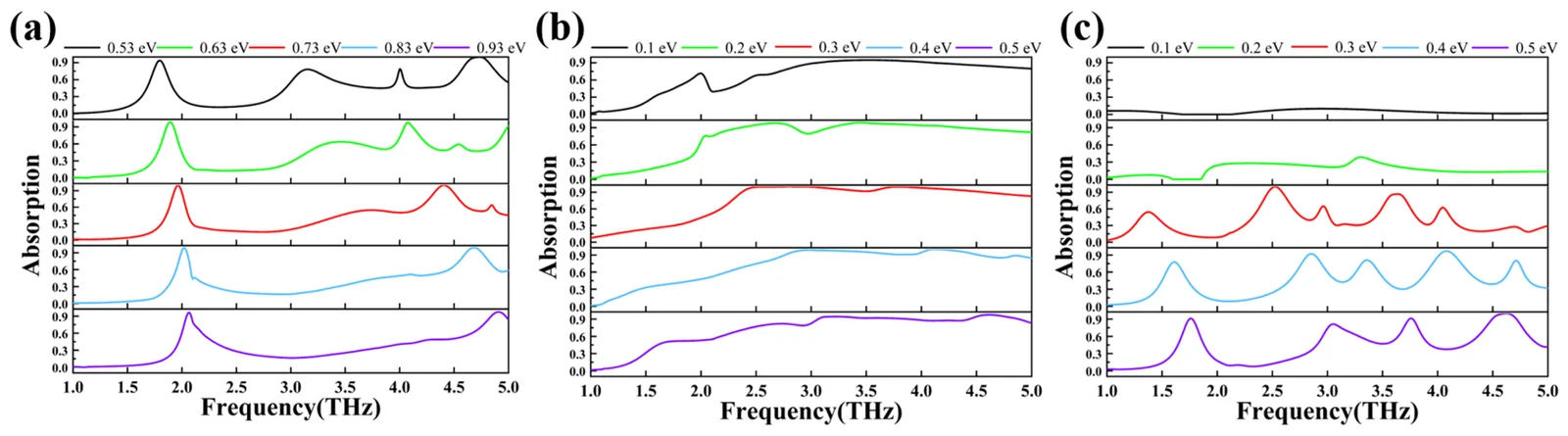
(**a**) Absorption spectra of Mode I under different Fermi levels. (**b**) Absorption spectra of Mode II under different Fermi levels. (**c**) Absorption spectra of Mode III under different Fermi levels.

As can be seen from Figure 10, for Mode I, via modulation of graphene’s Fermi level, we find that an increase in Fermi level induces a blue shift in the resonant frequency of the absorption peaks. In this regime, VO_2_ exerts negligible influence on the electromagnetic field and merely serves as a dielectric background. Accordingly, the tunability of the entire structure relies entirely on graphene. Varying the Fermi level directly modulates the equivalent surface impedance of graphene, while the dielectric background (VO_2_) remains unchanged. As a result, the resonant frequency varies monotonically with graphene parameters, realizing tunable control over the resonant frequency. For Mode II, as VO_2_ transitions to the metallic phase, adjusting the Fermi level of graphene broadens the absorption bandwidth under the ultra-broadband absorption condition at high temperatures. In Mode II, tuning the graphene Fermi energy while maintaining VO_2_ in the metallic state enables dynamic control of the absorptivity, resonance frequencies, and absorption bandwidth. This demonstrates the outstanding dynamic tunability of the proposed structure across all three absorption modes. This demonstrates that, under the idealized simulation conditions considered in this work, the proposed structure is tunable across all three absorption modes. It should be noted that this study is based on idealized electromagnetic simulations. In a practical device, local heating during the VO_2_ phase transition may affect the gate dielectric, electrical contacts, and graphene carrier mobility. For liquid or microfluidic sensing, heat transfer to the analyte may additionally introduce a temperature-dependent refractive-index variation through *dn*/*dT*. These effects are beyond the scope of the present model and should be considered in future multiphysics simulations and experimental studies.

**Figure 11:**
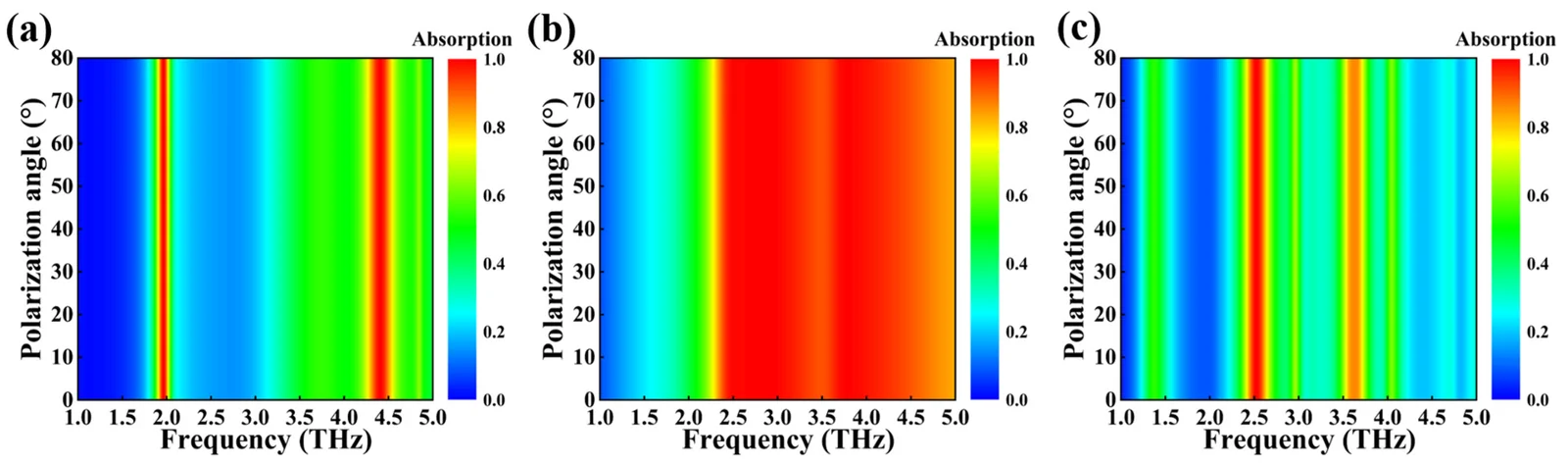
(**a**) Absorption scanning spectra of Mode I with varying polarization angles. (**b**) Absorption scanning spectra of Mode II with varying polarization angles. (**c**) Absorption scanning spectra of Mode III with varying polarization angles.

**Figure 12:**
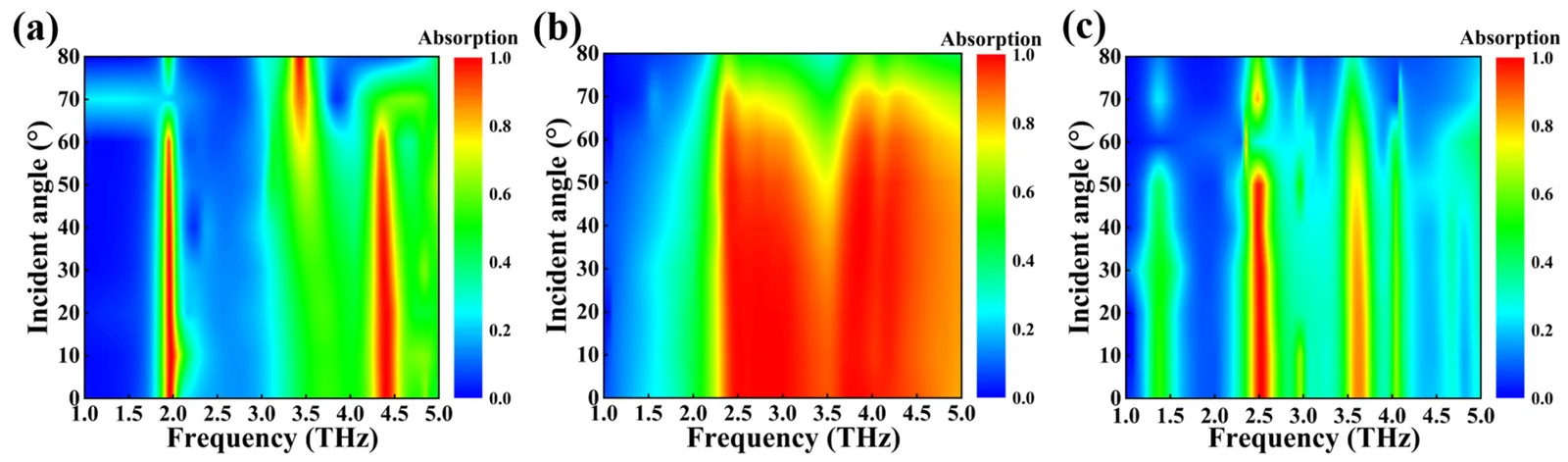
(**a**) Absorption scanning spectra of Mode I with varying incident angles. (**b**) Absorption scanning spectra of Mode II with varying incident angles. (**c**) Absorption scanning spectra of Mode III with varying incident angles.

Modes I, II, and III all exhibit good polarization insensitivity. Within the polarization angle range of 0°–80°, the absorption spectra of modes I, II, and III are shown in Figure 11a, Figure 11b and Figure 11c, respectively. It can be seen that all three absorption modes are insensitive to the polarization angle of the incident electromagnetic waves, and the absorption response does not change significantly with the polarization angle, indicating that the proposed absorber exhibits excellent polarization insensitivity in all operating modes. The absorption spectral response at different oblique incidences ranging from 0° to 80° is displayed in Figure 12. When the incident angle is restricted within the scope of 0° to 50°, the absorption performance remains stable, with the average absorptivity consistently maintained above 90%. For Mode I, the absorption peak pattern shows no significant change within incidence angles covering 0° to 60°. Yet when the incidence angle surpasses 60 degrees, all absorption modes vanish except a solitary peak located at 3.44 THz. This is because the increase in incident angle alters the effective area of the absorber exposed to electromagnetic waves, resulting in a pronounced change in absorptivity [74]. Similarly, the absorptivity of Mode II degrades at large incident angles, which is also attributed to the change in the effective interaction area between the absorber and electromagnetic waves caused by the increasing incident angle [75]. The same mechanism applies to Mode III. The degradation at large incident angles cannot be attributed solely to a reduction in the effective interaction area. Oblique incidence changes the in-plane wave vector and the wave impedance, thereby modifying the impedance matching, resonance frequency, and coupling between the incident field and the patterned layers. Moreover, TE and TM waves should be distinguished: for TE incidence, the electric field is perpendicular to the plane of incidence, whereas for TM incidence, the magnetic field is perpendicular to that plane. Their angle-dependent wave impedances and field components are different, so they may excite the resonant modes with different efficiencies. Although the fourfold symmetry supports polarization-insensitive behavior at normal incidence, TE and TM responses may diverge at large angles. Therefore, the observed deterioration should be understood mainly as the combined result of impedance mismatch, resonance shifts, and weakened mode excitation [76]. Overall, the proposed absorber features low sensitivity to wide incident angle variations, making it suitable for a broad range of application scenarios.

For the refractive-index sensing analysis, the analyte is assumed to cover the patterned graphene surface and occupy the superstrate region above the absorber. The refractive index of this superstrate is uniformly changed from 1.00 to 1.08, while the thicknesses and material properties of the five-layer absorber remain unchanged. In other words, the sensing environment is modeled by replacing the original surrounding medium above the graphene with a homogeneous analyte layer of variable refractive index. The analyte layer is assumed to be sufficiently thick compared to the evanescent-field penetration depth, so that the calculated response represents bulk refractive-index sensing rather than sensing of a finite-thickness molecular film. Under this assumption, the simulated resonance shift is determined by the interaction between the absorber’s near field and the entire analyte volume above the graphene. The sensitivity is calculated from the resonance–frequency shift per refractive-index unit, $S=\frac{\Delta f}{\Delta n}$, and the FOM is obtained by normalizing the sensitivity to the corresponding resonance linewidth [77,78]. This bulk-sensing model provides a reproducible basis for comparing the reported sensitivities of 0.25 THz/RIU and 1.75 THz/RIU in Mode I, together with the maximum FOM of 5.13 obtained in Mode III. For surface sensing, where the analyte forms only a thin layer on the graphene, the response would depend additionally on the analyte thickness and the near-field decay profile. Therefore, the present results should be interpreted as bulk or effective-superstrate sensing results, and a finite-thickness surface-sensing model would be required for quantitative analysis of molecular films or ultrathin analyte coatings.

**Figure 13:**
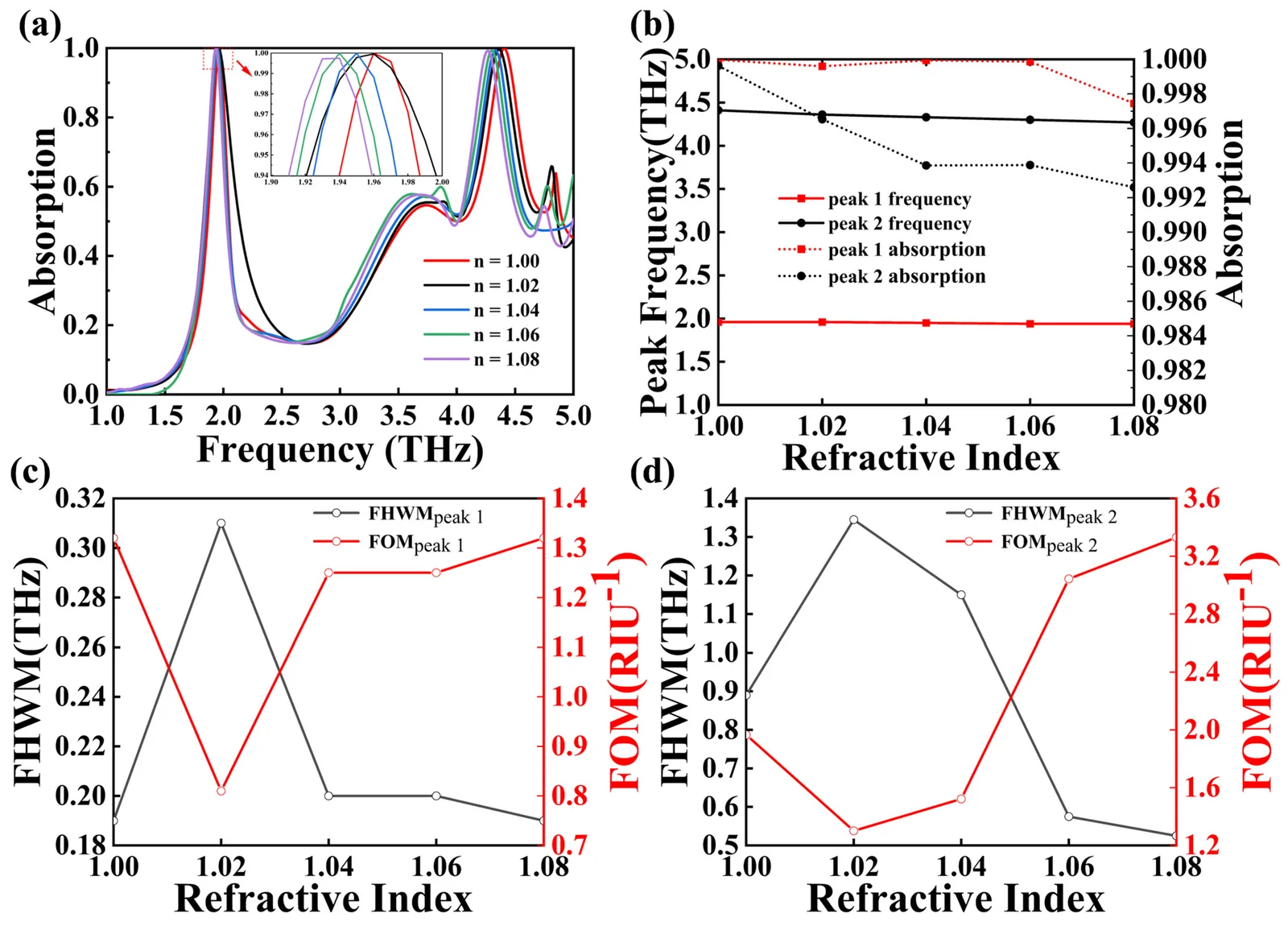
(**a**) Absorption spectra of Mode I for refractive index sensing with ambient refractive index varying from 1.00 to 1.08. (**b**) Frequency and absorptivity variations in two absorption peaks of Mode I for refractive index sensing. (**c**) Variations in FWHM and FOM at 1.96 THz for Mode I applied to refractive index sensing. (**d**) Variations in FWHM and FOM at 4.41 THz for Mode I applied to refractive index sensing.

**Figure 14:**
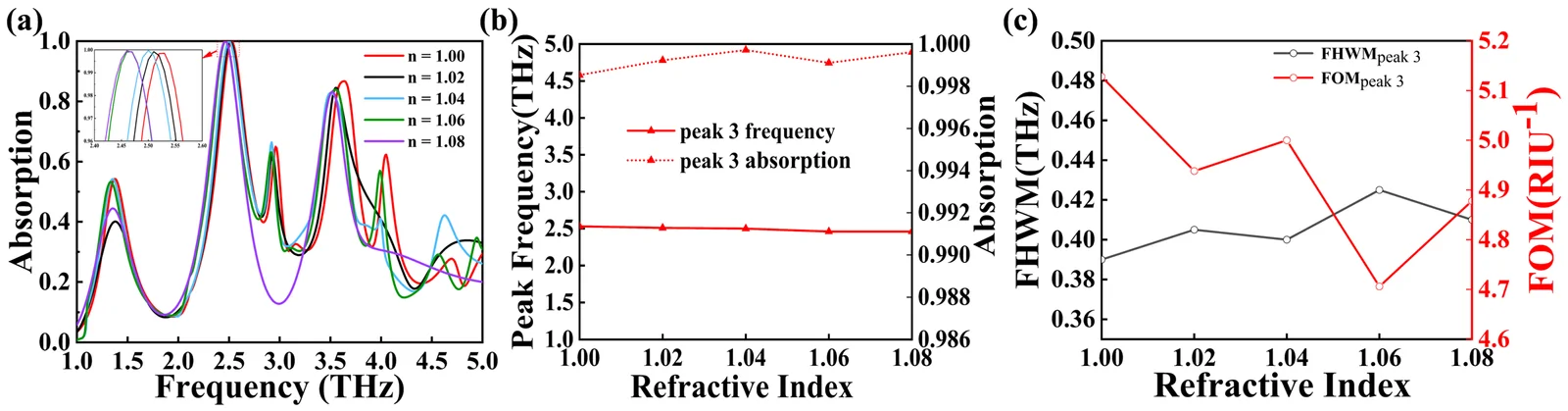
(**a**) Mode III absorption spectra for refractive index sensing with the surrounding refractive index tuned between 1.00 and 1.08. (**b**) Variations in absorption peak frequency and absorptivity of Mode III for refractive index sensing. (**c**) Variations in FWHM and FOM at 2.53 THz for Mode III applied in refractive index sensing.

As shown in Figure 13 and Figure 14, the refractive-index sensing performance of the narrowband resonances in Modes I and III is investigated by varying the ambient refractive index from 1.00 to 1.08. The interaction between the localized resonant fields and the surrounding dielectric medium modifies the effective permittivity of the resonant environment, resulting in a redshift of the absorption peaks. The sensing characteristics of these two modes are quantitatively evaluated in terms of sensitivity, Q-factor, and figure of merit (FOM). Under a refractive-index change in Δ*n* = 0.02, the corresponding sensing parameters are calculated as follows [77]:

(10) $$S=\frac{\Delta f}{\Delta n}$$

in this definition, Δf characterizes how much the absorption peak frequency shifts due to analyte refractive index fluctuations, whereas Δn quantifies the corresponding variation in the analyte’s refractive index. Over the investigated index interval, the 4.41 THz resonance in Mode I exhibits the highest sensitivity of 1.75 THz/RIU, while the other resonances show lower sensitivity values. Because the resonance shift can deviate from linearity as the surrounding dielectric environment changes, the sensitivities obtained here represent the average responses within the simulated range of *n* = 1.00–1.08. This range therefore defines the calibration interval for the sensing characteristics presented below.

To evaluate the sensing characteristics across different frequency bands, the figure of merit (FOM) is introduced for quantitative calculation:

(11) $$FOM=\frac{S}{FWMH}$$

The Q-factor characterizes the absorption peaks’ sharpness. For narrowband absorbers in particular, a higher quality factor contributes to enhanced sensing properties. The Q-factor adopted in this research is derived from the following equation [79]:

(12) $$Q=\frac{f}{FWMH}$$

For Mode I, the sensitivities of the resonances at 1.96 THz and 4.41 THz are 0.25 THz/RIU and 1.75 THz/RIU, respectively, with corresponding FOM values of 1.35 and 3.33. For Mode III, the resonance at 2.53 THz exhibits a sensitivity of 0.875 THz/RIU and an FOM of 5.13. These values characterize the average refractive-index responses over the simulated range of *n* = 1.00–1.08. In an experimental terahertz time-domain spectroscopy system, the measurable resonance shift can also be affected by source-power fluctuations, detector noise, finite spectral resolution, and phase instability. In addition, the reduction in peak absorptivity with increasing refractive index may decrease the resonance contrast and make peak identification less reliable when the signal-to-noise ratio is low. Therefore, the sensing functionality should be validated using repeated measurements and an appropriate spectral-fitting procedure rather than relying solely on the raw minimum of the absorption curve. The resonance linewidth and the uncertainty in the extracted peak frequency should also be considered when determining the minimum detectable refractive-index change. The present results are based on ideal electromagnetic simulations and demonstrate the intrinsic sensing response of the structure; experimental noise, fabrication tolerances, and system instability will need to be incorporated in future experimental validation.

At the conclusion of this work, we conduct a systematic comparative analysis between the proposed absorber and other absorbers reported in the literature, with the detailed results compiled in Table 4 [18,80,81,82,83,84,85]. It can be observed from the table that the comparison covers a variety of designs including broadband absorption, narrowband absorption, single-band, multi-band, and switchable broadband/narrowband modes, ensuring the comprehensiveness and impartiality of the comparison.

In Refs. [80,81,82], their Q-factors are marginally higher than that of our design, but they are significantly inferior to the present work in both sensitivity and FOM. In Refs. [83,84], although the switching between broadband and multi-band absorption modes can be achieved via tuning the external bias to enhance the functional integration and application flexibility of the device, neither of the two studies has further applied their structures to refractive index sensing, nor have they calculated the corresponding sensitivity parameters. Although the dual-band absorber reported in Ref. [85] boasts an extremely high Q-factor, its sensitivity is conspicuously low. Therefore, the absorber designed in this work exhibits a stronger responsiveness to variations in the environment under test, making it more suitable for sensing applications. The single-band structure proposed in Ref. [18] delivers moderate sensitivity when serving as a refractive index sensor, yet the design lacks dynamic tunability, which restricts the application scenarios of the absorber in practical use. By contrast, our design achieves a better overall balance among sensitivity, FOM, and dynamic tunability, while maintaining a competitive Q-factor. Overall, the absorber proposed in this work strikes a better balance among multiple performance parameters.

It should be emphasized that the devices summarized in Table 4 operate under different frequency ranges, sensing environments, structural configurations, and performance-evaluation conditions. Therefore, the comparison is intended to illustrate the functional characteristics of the proposed design rather than to claim absolute superiority in every sensing metric. Although some previous sensors report higher sensitivity or FOM values, the present absorber integrates dual-narrowband, single-narrowband, and broadband absorption modes within one tunable five-layer structure. The main advantage of this work is consequently the combined multi-mode switching capability and sensing functionality, rather than the highest individual sensitivity or FOM.

## 4. Conclusions

In this section, we propose a multi-mode switchable terahertz absorber with graphene and vanadium dioxide as core functional materials. The device supports three operating modes: dual-frequency narrowband absorption, broadband absorption, and single-frequency narrowband absorption. The peak absorptivity exceeds 99% under all three modes, which substantially improves the overall device performance. The electric field distributions of the absorption peaks in different modes are elaborated in detail, and the physical mechanisms underlying the generation and variation in each absorption peak are elucidated based on the impedance matching theory. We further systematically investigate the influence mechanisms of key parameters, including structural period, dielectric layer thickness, graphene Fermi level, polarization angle of incident light and incident angle, on the absorption performance of this type of device. Simulation results demonstrate that the proposed device maintains favorable stability within an incident angle range of 0° to 50° and a polarization angle range of 0° to 80°, and features the merits of incident angle and polarization insensitivity. The refractive-index-dependent responses of the narrowband resonances in Modes I and III are further analyzed over the range Δn = 1.00–1.08, demonstrating the sensing applicability of the proposed absorber. Finally, a comparison between this work and various previously reported absorbers indicates that the proposed device has notable advantages in terms of multi-mode switching capability and comprehensive sensing performance. The proposed five-layer structure is compatible with conventional microfabrication processes. A practical fabrication route may include deposition of the bottom gold layer and SiO_2_ spacer, deposition and lithographic patterning of the VO_2_ film, deposition of the upper SiO_2_ layer, and transfer and patterning of graphene on the top surface. Metal contacts for graphene gating and a microheater for VO_2_ thermal control can be placed outside the active resonant region. The principal challenges include maintaining uniform VO_2_ phase-transition properties, controlling the thicknesses of the dielectric layers, minimizing graphene transfer contamination, and achieving accurate alignment between the graphene and VO_2_ patterns. The present work is based on numerical simulations and does not yet include experimental validation. Experimental fabrication and electrothermal characterization will therefore be necessary to verify the predicted absorption performance and switching behavior.

## Figures and Tables

**Table 1:** Parameters of Patterned Graphene and Vanadium Dioxide for the Multimode Absorber.

Parameter	P	w1	w2	w3	w4	w5	w6	w7	w8	w9
Unit (μm)	22	6	5.4	12.8	3.6	0.5	6	5.6	12.8	4.1

**Table 2:** Layer Thicknesses of the Multimode Absorber.

Parameter	t1	t2	t3	t4
Unit (μm)	5	10.7	0.1	1.2

**Table 3:** Parameters related to different modes of the proposed absorber.

Mode	E_f_ (eV)	$\sigma_{{VO}_{2}}$(S/m)	Number of Peaks	Bandwidth (THz)	A_ave_
**I**	0.73	20	2	/	99.95%
**II**	0.3	200,000	/	2.21	96.62%
**III**	0.3	302.5	1	/	99.85%

**Table 4:** Comparison between the absorber designed in this chapter and previously reported absorbers.

Reference	Number of peaks	Tunability	Sensitivity [GHz/RIU]_max_	FOM_max_	Q-Factor_max_	Absorption_max_ [%]
**[80]**	1	Yes	984	3.57	14.54	99.99
**[81]**	1	Yes	114	3.15	11.20	99.5
**[82]**	1	Yes	500	3.85	13.23	99.21
**[83]**	Broad-band Narrow-band	Yes	/	/	/	Broad-band: >99 Narrow-band: >95
**[84]**	Broad-band Narrow-band	Yes	/	/	/	Broad-band: >80 Narrow-band: >90
**[85]**	2	Yes	398	/	Over 49	>99
**[19]**	1	NO	2100	7.03	6.8	>99
**This work**	3	Yes	1750	5.13	10.32	99.99

## Data Availability

Data underlying the results presented in this paper are not publicly available at this time but may be obtained from the authors upon reasonable request.
